# Experience of verbal violence among Chinese nursing students in clinical practice: a qualitative study

**DOI:** 10.1186/s12909-023-04741-z

**Published:** 2023-10-16

**Authors:** Meiyan Qian, Pingting Zhu, Qiwei Wu, Wen Wang, Guanghui Shi, Yinwen Ding, Hui Zhang, Xinyue Gu, Ting Xu, QianQian Zhang

**Affiliations:** 1 School of Nursing, School of Public Health, Yangzhou University, 136 Jiangyang Middle Road, Yangzhou, China; 2 Jiangsu Key Laboratory of Zoonosis, Yangzhou, China

**Keywords:** Chinese, Nursing students, Qualitative research, Verbal violence, Workplace violence

## Abstract

**Background:**

Workplace violence is prevalent in the nursing profession, and as a relatively junior link of the professional hierarchy, nursing students are not immune to it. Among these, verbal violence may have more serious consequences for the victims than physical violence, but the literature on verbal violence among nursing students in Chinese clinical settings is limited.

**Aims:**

To explore the verbal violence experience among Chinese nursing students in clinical practice, and the strategies used by nursing students to cope with violence.

**Design:**

A descriptive qualitative study.

**Methods:**

From January 2022 to June 2022, semi-structured interviews were conducted with 21 nursing students in clinical practice by purposive snowball sampling. Nvivo12 software and inductive content analysis were used for data analysis. This paper followed the COREQ (Consolidated criteria for Reporting Qualitative Research) guidance.

**Results:**

Through data analysis, three themes were defined:(1) Multiform verbal violence; (2) Hurting and impacting and (3) Struggling or Coping. The findings indicated that nursing students were subjected to multiple forms of verbal violence in clinical practice, not only from patients and caregivers, but also from peers such as clinical tutors and doctors, which not only harmed students’ personal health and well-being, but may also contribute to the nursing industry’s future loss of human resources. Seeking emotional support from others and forcing themselves to grow up were the most commonly used coping strategies.

**Conclusion:**

Nursing educators and nursing managers need to pay attention to verbal violence in the clinical environment, and actively develop the ability of nursing students to deal with uncivilized behavior. Establishing relevant courses and training such as communication, resilience, and violence prevention, establishing a stricter clinical mentor appointment system, and teaching assessment system may be strategies to help nursing students better perform clinical practice.

## Introduction

Workplace violence against health care professionals is now recognized as a global public health issue. Nurses are on the front line of patient care and are required to spend more time in close contact with patients or visitors. Compared to other workers in healthcare settings, nurses face a greater likelihood of exposure to violence [1]. Available evidence shows that the type of violence against nurses varies by setting and region of the world, with rates ranging from 24.8 to 80.8% [2–4], but studies consistently show that verbal violence is the most common type of violence in healthcare settings [5, 6]. Unfortunately, as a group in a clinical setting, nursing students often witness and/or encounter verbal violence, which researchers believe is deeply ingrained in the nursing culture, but is largely unrecognized by students [7, 8].

## Background

Workplace violence remains problematic and highly prevalent in healthcare, with verbal violence being one of the most common types [5, 9]. In this study, verbal violence is defined as the use of abusive, mocking and other uncivilized language that causes mental and psychological pain or harm to others [10]. Although “zero tolerance” policies have been adopted in medical settings in many countries, most nurses are still victims of violence [11], while nursing students are more vulnerable. Studies have shown that 50.3% of nursing students reported experiencing violence due to many reasons such as low educational attainment, lack of confidence in nursing students, and dissatisfaction with treatment outcomes by patients or their caregivers [12, 13]. In addition, 51.23% of nursing students experienced vertical violence including criticism by injustice, public humiliation, with 46.6% of events originating from clinical nursing teachers, 39.4% from doctors, and 21.3% from nursing managers [14]. Hence, they face violence not only from external personnel but also from internal personnel, which is an important issue for nursing students who are at the junior level of the hierarchy in the profession.

Nursing education consists of a theoretical and practical component, with the practical component providing students with the opportunity to apply theoretical knowledge from the classroom to clinical practice. In China, nursing students are required to complete a minimum of eight months of clinical practice in their final year of university, with practice arrangement covering almost all departments. Studies have shown that emergency medicine, pediatrics, and psychiatry are high-risk departments for violence, with incidence rates ranging from 55.5 to 97.6% [15, 16], while nurses working on a shift pattern are more vulnerable to violence compared to nurses on fixed duty, especially those on night shift [17]. Yet nursing students are called to implement the same shift system as registered nurses, and they are obliged to work in potentially high-risk areas early in their careers. Meanwhile, since nursing students are in the process of transitioning from school to society, as well as their relative lack of social and job experience, they are vulnerable to becoming victims of verbal violence [18].

Worldwide, verbal violence among nursing students is becoming increasingly common [18, 19]. A study in Turkey found that 91.1% of nursing students suffered verbal violence in clinical practice [20]. In the UK and Australia, well over half of students reported experiencing or witnessing significant non-physical violence during their clinical rotations [19, 21]. Yelling, threatening, shouting and name calling were the most commonly reported forms of verbal violence [22]. A qualitative study in Turkey found that medical professionals would devalue students’ approach to patient care, and stigmatize and treat students as less valuable medical professionals [23]. However, nursing students tended to be reluctant to report incidents of violence, so the actual incidence of violence may be underestimated [24].

Unsurprisingly workplace violence has a significant negative impact on nursing students. Nursing students reported experiencing physical problems including headaches, diarrhoea and fatigue [25], while experiencing violence can have adverse emotional consequences such as stress, anger, embarrassment, despair and even post-traumatic stress disorder [19, 26]. These emotions can be detrimental to the learning experience in that the clinical environment becomes a source of distress rather than a valuable and rewarding professional experience [12]. Studies have found that verbal violence was less frequently reported than physical violence due to the insidious nature of the harm it can cause [24, 27]. In some cases, however, verbal violence has been reported to bring about the same degree of psychological distress or even worse repercussions for the victim as physical violence [28].

China is a developing country, and its medical and health systems have not yet reached developed-country standards. Patients are frequently frustrated because they are unable to resolve the defects in the medical and healthcare systems, and they tend to vent on the medical staff. Organized medical harassment, known as “Yinao” in Chinese, also exists in China, with a high incidence of violence [4], which has led to a high level of fear of future violence in the workplace and a high turnover of nurses [29].

According to a recent review of the literature, verbal violence against nurses occurs in 63% of Chinese healthcare settings, with more incidents occurring in economically developed provinces like Beijing and Jiangsu than in less developed ones [30]. Younger nurses are also more likely to be victims of verbal violence. However, research has mainly concentrated on registered nurses [4, 16, 31], and there is little information available about how verbal violence is experienced by nursing students in clinical settings. Considering that nursing students have to adapt to their new role, they may be afraid to speak up about unfair workplace treatment. At the exact same time this group acts as a back-up for nursing human resources and affects the stability of the nursing profession, therefore it is essential to focus on nursing students’ experiences of verbal violence in clinical practice and provide strategies to better cope with this problem [32]. The purpose of this study was to explore the experience of verbal violence among Chinese nursing students in clinical practice and the strategies used by nursing students to cope with violence. It is hoped that this paper will provide actionable and constructive suggestions for the prevention and control of verbal violence among nursing students.

## Methods

### Study design

Descriptive qualitative research was used to resolve these research questions: (1) What are the experiences of Chinese nursing students with verbal violence? (2) What are the impacts of verbal violence on nursing students? and (3) What are the strategies for nursing students to deal with verbal violence? This approach, which used internal narratives to obtain a rich and direct description of the experience, perception, or event of interest [33], is suitable for examining healthcare and nursing-related phenomena and is particularly useful when the phenomenon of interest is not well known [34].

### Participants

The purposive and snowball sampling method was adopted to recruit graduating nursing students at four hospitals in Jiangsu Province and one in Shanghai. Inclusion criteria for participants included:(1) age ≥ 18 years; (2) internship duration ≥ 6months; (3) exposure to verbal violence. Ultimately, we included 21 participants. The sample size was determined by data saturation, that is, no new topics were identified in three consecutive interviews [35].

### Data collection

In this study, one-to-one, semi-structured interviews were conducted in offline or online ways. The research team (MY Q, WW, SGH) constructed an interview outline by reviewing literature on violence [36, 37]. Following pre-interviews with two nursing students, the outline was adjusted and then a formal interview outline was developed through group discussion. The final interview outline included:

(1) Can you briefly describe the most memorable or most recent experience of verbal violence you have had?

(2) What problems did you encounter after the experience of verbal violence? How did you cope with the verbal violence?

(3) Why do you think nursing students are subjected to verbal violence?

(4) Has verbal violence had any impact on you?

(5) Do you have any suggestions for reducing verbal violence?

Offline interviews were conducted in a quiet, undisturbed room (conference room or office) and online interviews were done via WeChat or telephone call, all interviews were conducted in Chinese. No one else was present besides the participants and the researchers. Interviews were conducted by two people (MY Q & QW W), they were both female nursing students with an MSc in nursing and their research direction was nursing education. Prior to the interview, the researchers informed the participants of the study objectives and procedures, and established a trusting relationship with the participants by exchanging pleasantries before the interview. The researchers utilized the interview outline as a guide throughout the interview, along with exploratory questions to allow participants to express their own viewpoints, and modified the interview’s sequence and content to suit the circumstances. The interview process was based on listening carefully to the interviewee’s point of view, and the process was recorded and transcribed after obtaining the participant’s consent. To ensure the completeness and accuracy of the information, the interview recordings were listened to in pairs within 24 h of the end of the interview, and the content of the recordings was converted verbatim into textual information. The data collection period was from January 2022 to June 2022, and each interview lasted 21–57 min.

### Data analysis

Data was analyzed using inductive content analysis to present themes and sub-themes and reported using the Consolidated criteria for reporting qualitative research (COREQ) [38–40]. The transcribed interview data was imported into the qualitative analysis software Nvivo12 and the data was analyzed by two researchers (MY Q & QW W). Themes emerged from the data rather than deductively obtaining them from pre-existing themes in the literature. The analysis process was divided into four main steps [41]: First, the researchers repeatedly read and immersed themself in the material to gain a sense of the whole. Next, phrases that answered the aims were selected to be meaning units, either explicit or implicit, then the condensed meaning units were open coded. According to their common properties and meaning, codes were reviewed and organized into category units. Third, naming and defining initial categories, the category was named using words/phrase that best represented the category. Constant comparisons were utilized to ensure there was no overlap between categories. Finally, the categories were organized according to their common attributes to form a sub-theme. Differences were resolved through discussion and group consensus. The analysis process was conducted in Chinese, and after the themes and exemplary quotes confirmed, they were translated into English by PT Z and MY Q who were fluent in English. The back-translation of this text was done by another researcher (QW W) who was proficient in Chinese and English to check the accuracy of the translation, and after a final comparison and discussion, the final translation was created.

### Rigor and reflexivity

The rigor of results was assessed in terms of credibility, transferability, dependability, and confirmability to develop the trustworthiness of research [42]. Credibility: Researcher returned the results to all 21 participants online to ensure that the findings accurately represented what they want to say. Transferability: Through the detailed description of the methods, the transferability was ensured. Dependability: Researchers had extensive experience in qualitative research and the soundness of the research protocol was assessed by an external researcher. Confirmability: Besides member checking, confirmability was achieved through data triangulation. Accuracy in the data collection and analysis process was optimized through both field notes and interview transcriptions to avoid or minimize errors or biases. Qualitative research must be reflective, the researchers needed to recognize and acknowledge their own biases and prior assumptions about verbal violence and provide space for participants to share their experiences.

## Results

Twenty-two individuals were approached to participate, ultimately 21 nursing students participated in the qualitative interview study with one student withdrawing from the study due to lack of time. Three of them were males, the other 18 were females. Participants ranged in age from 21 to 24, with average clinical practice duration of 7.09 months. (Table 1, at the end of the document text file)

**Table 1:** Demographic Characteristics

Participant No	Age (years)	Gender	Education degree	Recruitment Avenue ^a^	Duration of clinical practice (months)	Current clinical department
P1	22	Male	Undergraduate	KSPH	8	Psychiatry
P2	22	Female	Junior college student	WXSPH	6	General Surgery
P3	22	Female	Undergraduate	SHCH	8	Emergency department
P4	22	Female	Undergraduate	KSPH	8	Psychiatry
P5	21	Female	Undergraduate	NJPH	6	Obstetrics
P6	22	Female	Undergraduate	KSPH	8	Psychiatry
P7	22	Female	Undergraduate	AHYZU	8	Gastroenterology
P8	21	Female	Junior college student	AHYZU	7	Orthopedics
P9	22	Female	Undergraduate	AHYZU	6	Urinary Surgery
P10	22	Female	Undergraduate	WXSPH	9	ICU
P11	22	Female	Junior college student	AHYZU	8	Neonatal
P12	22	Female	Undergraduate	SHCH	6	Neonatal
P13	21	Male	Undergraduate	AHYZU	6	Neurology
P14	22	Female	Undergraduate	NJPH	6	Paediatrics
P15	22	Female	Undergraduate	NJPH	6	Military Sursery
P16	22	Female	Undergraduate	NJPH	6	Neonatal
P17	22	Female	Undergraduate	NJPH	6	Orthopedics
P18	24	Female	Junior college student	WXSPH	8	ICU
P19	22	Female	Undergraduate	AHYZU	9	Gastroenterology
P20	21	Male	Junior college student	SHCH	6	Cardiology
P21	23	Female	Undergraduate	KSPH	8	Neurology

^a^ KSPH: Kunshan People’s Hospital; WXSPH: Wuxi Second People’s Hospital; SHCH: Shanghai Children’s Hospital; NJPH: Northern Jiangsu People’s Hospital; AHYZU: Affiliated Hospital of Yangzhou University

**Fig. 1:**
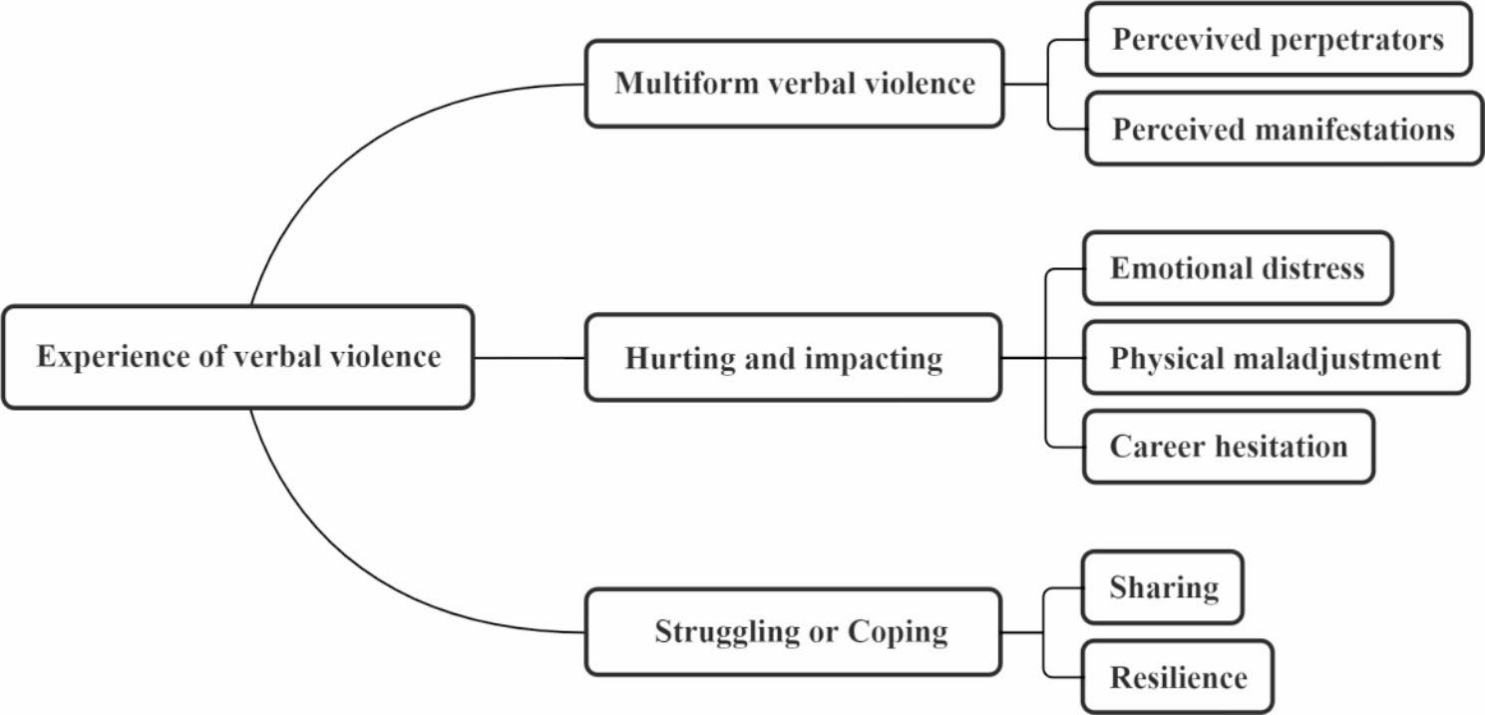
Themes and sub-themes identified from in-depth interviews on experience of verbal violence

Through data analysis, three themes were defined: (1) Multiform verbal violence; (2) Hurting and impacting and (3) Struggling or Coping (Fig. 1).

### Multiform verbal violence

#### Perceived perpetrators

Patients and caregivers were the most commonly described perpetrators of violence, and violence could occur in regardless of ward, outpatient, or emergency department visits. Participants believed that illness sensitized patients and caregivers, and that nursing students, who were at the bottom of the clinical environment, easily became an outlet for patients’ and caregivers’ emotions.

*“I thought it was because they (caregivers) were too anxious about their family members or they (patients) were too addicted to the role of the patients, so they were easy to be angry and shout at us” (Participant 15)*.

Clinical tutors were also frequently mentioned perpetrators of violence, and participants perceived clinical tutors as senior as well as experienced care providers to highlight work status by implementing intentional and unintentional verbal violence against nursing students. Additionally, participants thought clinical tutors had to deal with disappointments in their personal life in addition to work-related ones, consequently they saw nursing students as “breathing buckets” for them to let out their negative feelings.

*“I thought it was a way to for them to let out negative feelings, but again, I thought it was a very bad catharsis for them to take it out on the students.” (Participant 15)*.

Although they were mentioned sparingly, institutional nurses and physicians were equally regarded as verbally violent perpetrators. Participants said they rarely had the opportunity to work with them because they always followed their clinical tutors, and the operating room was where they suffered the most verbal violence from doctors.

*“The professor was so fierce that I forgot to hand him the right scissors, and then he immediately reprimanded me.” (Participant 7)*.

#### Perceived manifestations

Nursing students were “new” to the clinical environment, and patients or caregivers may question the competence of nursing students. Participants said that when they attempted to clear up patients’ doubts, the patients and their caregivers questioned the veracity of their responses and sought the advice of another doctor. Participants perceived this as a slight and distrust of their abilities, which was psychologically damaging.

*“I explained the precautions to him, but he asked the doctor again, obviously our answers were the same, even we answered more carefully, I was speechless.” (Participant 10)*.

Being shouted at or yelled at was the type of verbal violence that almost all participants experienced. Nurses were understaffed despite the large amount of patient care. Participants claimed that they never took a break but suffered shouting or yelling from patients or caregivers as a result of not being able to quickly alter every patient’s medicine. Additionally, despite their best intentions to offer their patients with high-quality nursing care, nursing students occasionally caused suffering, which resulting verbal violence. Participants wanted these perpetrators to empathize and give them more tolerance.

*“When I was busy, she suddenly ran to stop me, just shouted at me in the hall, asking me why I didn’t come, asking me why I didn’t treat her child properly.” (Participant 11)*.

*“They can be a little more tolerant of that newbie, and then they can choose a slightly tactful way when expressing it.” (Participant 2)*.

Due to the requirements of COVID-19 prevention and control, a patient could only have one companion (wearing a dedicated wristband), and no one else could enter the hospital to visit the patient. Nursing students were sometimes placed at the entrance to the ward to screen incoming and outgoing visitors to prevent anyone from entering the ward who was not qualified as an escort. When the demand for visits (especially visits to dying patients) was not met, some people got angry and verbally attacked nursing students.

*“They called me a psycho and said I don’t let them in, and they kept cursing there.” (Participant 2)*.

Participants also described their experiences of being shouted at by clinical tutors for no apparent reason, which they found incomprehensible. The participants believed this was due to the lack of standardized training of tutors and hoped that the hospital could strengthen the training of clinical tutors.

*She was probably in a bad mood that day and kicked that bucket over as soon as she came in. Just yelled at me, but the truth was I didn’t do anything. (Participant 11).*

Patients and relatives tended to focus their own attention on the disease and thus would overlook things. When something went wrong, they didn’t admit it was their fault. Instead, they put the blame on the nursing students. In the minds of patients or relatives, poor skills were the inherent impression of nursing students. As a result, when registered nurses made mistakes, they would automatically regard the pain as brought by students and complained to relevant departments after discharge, and nursing students became the “scapegoat”.

*“He couldn’t find his escort card …I was the one who handled it for him… He said I was irresponsible and didn’t admit to taking his things.” (Participant 8)*.

Clinical tutors sometimes shirked responsibility to nursing students. Clinical tutors occasionally refused to acknowledge their mistakes when nursing managers found them, claiming that they had already handed the work to the students in order to avoid reprimands and punishment. Examples of these mistakes included not filling out the record book and failing to promptly clean up the treatment tray.

*“The nursing managers asked why the record was blank…My clinical tutor questioned me in front of the nursing managers, questioned me three times, and said that she had taught me, and I didn’t remember it.” (Participant 9)*.

### Hurting and impacting

#### Emotional distress

Nursing students showed a variety of diverse emotional reactions when exposed to various violent scenarios. They expressed anger and grievance when dealing with unjustified or unreasonable verbal violence from patients or caregivers and felt disrespected. At the same time, in the face of verbal violence from their clinical tutors, participants indicated that as clinical tutors they should be friendly to their students and patient in teaching knowledge and skills. In clinical practice, the tutor should act as a protector, not an abuser, and participants often felt angry and aggrieved about this.

*I felt very angry, you (clinical tutor) did this, obviously you were wrong, why you treated me like this? (Participant 13).*

Faced with verbal violence by patients and caregivers, participants showed high empathy as well as understanding. They believed getting disease was an unfortunate thing, that not only caused physical harm to patients, but also affected the mental health of patients and caregivers, through the implementation of verbal violence to others was a way to release their pain. In Chinese culture, individuals focused on the health of their families that they wanted patients to be adequately treated and cared for. Patients also typically had larger needs for safety and love. When their demands weren’t met, they lost control. In this case, participants put themselves in the patient’s or caregiver’s shoes to understand why they were committing violence. However, when exposed to traumatic situations for a long time, some participants experienced emotional exhaustion.

*But they, because of the Chinese tradition, when someone in the family was sick, a big group of people must come to visit him/her, and they must be unhappy to be stopped at the door, which I thought was understandable. (Participant 14).*

*“I wanted to comfort them, but you know what, after being scolded so many times, you felt very tired and it was hard to empathize with each patient.”(Participant 4)*.

Participants described scenes where they were verbally abused in public and said that the loud abuse attracted the attention of many people around them. They felt so humiliated that they wanted to run away from the scene and were afraid that the patients would lose trust in them.

*“She was swearing at me in the corridor, I felt like everyone was looking at me and I felt so humiliated. How am I going to give care and do work for people when I am on this ward in the future?” (Participant 16)*.

#### Physical maladjustment

The consequences of the violence described by the participants in this study were sometimes distressing, and verbal violence had serious physical effects on them. Participants reported that verbal violence affected their state after work and that leaving work was no longer an enjoyable event. They tended to lose their appetite and did not want to eat anything, even when faced with their favourite food.

*“I couldn’t eat when I thought about it…Hotpot, that’s one of my favourite foods, but I really didn’t have much of an appetite that day, I was so angry.” (Participant 7)*.

Similarly, participants reported that scenes of violence suffered at work would suddenly come to mind and they would keep recalling the incident and they would toss and turn in bed, unable to get to sleep quickly. They even dreamed of scenes in which the patient was in conflict with themselves.

*“I would keep thinking about it…lying in bed and all of a sudden in a flash I remembered what that person said to you, that’s the kind of personality I had and then I had sleeplessness.” (Participant 19)*.

#### Career hesitation

Verbal violence caused nursing students work-related difficulties as well as psychological and physical damage, which may have caused them to reevaluate their career choices. The nursing profession was an overload, high pressure, and high-risk profession, and huge work pressure made nursing students used as manpower to reduce their own labor load by clinical tutors during clinic practice, and they were often scheduled to do some non-therapeutic nursing work such as measuring vital signs and changing liquid medicines. Participants reported that verbal violence severely demotivated them and that their productivity decreased. During this period, participants displayed a ‘retaliatory’ behaviour in which they perceived themselves as a helper to the tutors rather than a machine to be used at will.

*“I became very dull, and when I met the clinical tutor who told me I was stupid, I didn’t want to help him…And then I won’t volunteer to help him anymore.” (Participant 10)*.

Nursing students undoubtedly felt unfamiliar and afraid when they first entered the clinical setting because there were little opportunities for practice while they were in school, particularly when they had to administer treatments to patients. Being exposed to verbal violence caused participants to have doubts about their abilities, decreased confidence in caring for patients, and questioned their suitability for the nursing job.

*“I might think about whether I am fit to just be in nursing as a profession and I might also think about whether I am stupid and bad.” (Participant 13)*.

Many participants admitted that entering clinical practice was not their first choice and that they would leave nursing immediately if given the chance. A few participants also made it apparent that they would leave the nursing field because they felt that it was too stressful and eventually led to psychological trauma.

*“Sometimes when I was tired, I thought about whether I wanted to stick to the job.” (Participant 9)*.

*“Verbal violence caused psychological trauma, I think it was very hard to heal, and it was the kind of thing that you took a long, long time to heal it, even when you, you, you felt like you’ve gotten better, it was not.” (Participant 15)*.

### Struggling or coping

#### Sharing

We found that participants tended to remain silent after violence occurred rather than clash with the perpetrator, they sought support by sharing their encounters with others. When the perpetrator was not the tutor, they sometimes sought comfort by reporting the situation to the tutor.

*“I was angry that day, but I talked to my tutor, and then she helped me ease it.” (Participant 19)*.

Friends were the support systems most mentioned by participants. Participants indicated that most of the friends around them were in the same profession and were also in clinical practice, suggesting that they may have shared similar experiences, were more able to relate to them, understand their feelings, and offer support and guidance.

*“Sometimes (I) chose to find my friends in the same specialty, and then we shared the bad experiences with each other, and then (I) felt good, and then (we) ate a meal or something, it did not sound so difficult.” (Participant 13)*.

Parents were the strongest backing of their children, so after encountering violence, participants chose to reach out to their parents to express their inner pain and desired comfort and encouragement.

*“They were supportive and felt I was right, and then told me to go ahead and do this job without any worries.” (Participant 11)*.

#### Resilience

The participants felt that verbal violence was inevitable, although it was harmful to them, but they had to stick to clinical work in order to complete the internship successfully. During this period, they could only reduce or adapt to the occurrence of verbal violence by constantly improving themselves.

Participants reflected after having experienced violence and reported that they were aware of the importance of communication. Participants felt that effective communication could reduce the incidence of verbal violence to some extent. In many cases, they no longer chose to remain silent, but began to try to communicate with the patients or the clinical tutors and learned to master communication skills.

*“It’s possible…At the same time reminded us to pay more attention to communication ability and ease the relationship between relatives…It took skill to communicate with tutors and patients.” (Participant 5)*.

The participants realized that lack of solid theoretical knowledge and lack of good practical skills were significant causes of verbal violence. Despite the education they had gotten in school, they did not have enough chances to put it into practice. Upon entering the clinical settings they would be subjected to verbal violence because they were unable to meet the expectations of patients or clinical tutors. They determined to spend more time to improve themselves.

*“Of course, you had to master the theories and operations that your tutors taught you. People scolded you, but it was really your own bad work. What could you do about it? It’s that you try to improve yourself.” (Participant 19)*.

Participants believed that they were powerless to alter the beliefs and behaviors of others and that the only way to cope with the violence was to modify their own mentality. On the one hand, participants believed that anger was physically harmful. On the other hand, participants thought that they should not be regarded seriously because they were merely “passers-by” in their life and would not subsequently come into contact.

*“Sometimes I got angry, but I adjusted myself because I thought it’s not good for me physically.” (Participant 15)*.

## Discussion

This study confirmed that verbal violence was common in nursing student clinical practice, not only reflected in the wide coverage of perpetrators, but also reflected in the diverse forms of violence. Patients and caregivers were identified as the most frequent perpetrators of verbal violence against nursing students, followed by institutional nurses, and physicians [13], which was similar to our findings. Differently, clinical tutors were also described as violent perpetrators at times, which were rarely mentioned in other studies [43, 44]. Swearing, yelling, and rude language were the most commonly reported forms [44], which were similar to our study. The difference was that because of their “novice” status, nursing students received more questions from the outside, not only operational skills, but also knowledge reserves. As a relatively junior link of the professional hierarchy, nursing students are sometimes treated as scapegoats, possibly because they are more likely to be bullied and less able to resist. However, sometimes these behaviors have hidden nature, which makes it impossible for students to judge whether they can be described as violence, but the cumulative effect of these behaviors will cause students to feel powerless in the clinical environment and have a negative impact on them [45].

One of our new findings is that COVID-19 appears to be a contributing factor to the occurrence of verbal violence. Chinese regular management required checkpoints at the entrance to the ward as well as restricted visit (one person only) to reduce the risk of spreading infection. However, due to the factors of disease, patients’ families are often in a state of anxiety and fear [20], they are eager to visit patients to express their concern and relieve their tension. Hence, when nursing students counseled or stopped them from entering the ward, they tended to be unable to understand and feel angry. They were prone to verbal violence against nursing students, the incidence of violence rose significantly [46].

When confronted with verbal violence, nursing students displayed complex emotions, such as anger, anxiety, helplessness, self-blame and fear [13, 47]. However, we have found that nursing students often expressed anger and aggression when confronted with violence from medical staff. This mismatch in expectations will cause nursing students a tremendous deal of suffering because they frequently perceive the clinical tutor as a support system or “back wall” and defender of their rights [48], whereas in reality the clinical tutor is the one who attacks them. In recent years, due to the tense nurse-patient relationship, hospital managers have been more strict with nurses, which further aggravates the pressure of nurses and leads to the exhaustion of physical health and emotional state [49].Thus nurses may vent their negative emotions on nursing students, for instances failing to patiently provide adequate and clear instructions to students or even committing verbal violence against them [14]. Clinical practice in China is often based on a “one-on-one” teaching model, yet clinical tutors are appointed without a rigorous selection process and are not educated to supervise students, which results in registered nurses who may not be fully qualified to serve as clinical tutors. This suggests that nursing managers need to fully consider the needs of students when appointing clinical tutors, not only examine the work ability of registered nurses. At the same time, formal training should be carried out to enable clinical tutors to better assume the roles of mentor, supporter and protector. Finally, an assessment system can be set up to evaluate the work of clinical tutors on a regular basis, and the assessment situation can be included in the promotion of professional title, year-end evaluation and other contents.

We found that nursing students’ emotions were more complex when confronted with verbal violence from patients or their caregivers. They tended to be angry in response to their unreasonable verbal violence, but also showed a high level of understanding and empathy for verbal violence by patients and their relatives. Because they realized that the violence of patients may be the result of specific diseases, patients become more sensitive and have higher safety needs because of diseases [50], while the violence of relatives is caused by the concern of patients [51]. Empathy plays an important role in nurses’ daily work. A high level of empathy can help reduce medical disputes and relieve patients’ negative emotions [52], while a low level of empathy will make patients and relatives dissatisfied, and even lead to nurses facing a higher probability of verbal violence [4]. Nursing managers could conduct regular symposium focusing on the empathy level of nursing students to reduce the likelihood of violence against them. Clinical case-studies and reflective discussion were effective means of increasing nursing students’ empathy [53].

To a large extent, verbal violence also affected the physical health of the nursing students, who in this study described their experiences of insomnia and loss of appetite. In a study in Turkey, 62.2% of nursing students reported headaches and 26.2% reported sleep difficulties [20]. Students in New Zealand described their experiences in terms of a racing heart and distorted stomach [45], and binge eating, nausea, weight loss, and menstrual disorders have also been reported in the literature [54, 55]. The suffering caused by verbal violence is clearly enormous for some who experience or witness it.

Nursing students who experienced verbal violence were tormented in the profession. Similarly, in a study in Taiwan, violence was described as a “constant nightmare” and frequent exposure may severely reduce nurses’ professional attitudes and undermine their enthusiasm for the profession [56]. Nursing students also reported that these negative experiences affected their confidence and self-esteem, and made them question whether they were competent enough and had made the right career choices [45]. A surprising finding was that some nursing students would retaliate against their clinical instructors by neglecting or delaying the completion of assigned tasks. Similar to this finding is the tendency of some nurses who are victims of violence to retaliate by providing inadequate services to patients [57], which is detrimental to the quality and safety of care [58]. Nursing managers should be aware of this phenomenon and work to prevent its consequences.

The inevitable traumatic events in the work force nursing students to develop themselves to cope with the challenges. Put another way, verbal violence provides opportunities for nursing students to learn and improve their personal skills and clinical practice skills, but this may require nursing students to have strong psychological resilience [59]. Studies have found that nursing students with high resilience can better self-regulate, and they are good at learning lessons from hardship. They tend to adapt to stressful work in a positive way and better cope with violence [14]. Consistent with previous studies, improving communication ability and enhancing professional knowledge and skills are effective strategies to prevent verbal violence [60, 61]. Therefore, specific subjects related to communication, resilience, and violence should be included in university courses. Meanwhile, the health and education sector need to create a collaborative environment to promote training on violence prevention. Nursing educators can try to integrate violence education into their course instruction by using simulated scenarios or role plays to show students what verbal violence is, how it arises, and how to respond to it, while real cases of verbal violence can be collected for classroom discussion. And nursing managers can enhance nursing students’ verbal violence prevention practice skills using formats such as hospital violence sharing sessions. In addition, for the nursing students themselves, they should learn enough knowledge and skills to constantly improve their abilities, because this can usually prevent violence against them.

### Limitations

First, the settings of participants’ internship were only performed in tertiary hospitals, and the experience of nursing students in primary or secondary hospitals may be different, which was beyond the scope of this study. Second, the study asked participants to recall past experiences, so recall bias could be a factor.

## Conclusion

Chinese nursing students generally experience verbal violence in clinical practice, which not only affects the physical and mental health of nursing students and the quality of work, but also hinders the construction of health care workers. Therefore, nursing educators and nursing managers need to pay attention to the issue of verbal violence. Nursing educators should provide nursing students with high-quality education on violence and offer courses to guide students to improve their professional theoretical knowledge, practical skills, communication skills, and psychological resilience. Nursing managers should promote an active practice environment, including strict clinical tutor appointment system and teaching assessment system, while nursing students should be trained in clinical practice to help them better cope with violence. The implications of this work provide information on the development of education, practice culture and policy, and provide a basis for constructing verbal violence interventions for nursing students.

## Data Availability

The datasets used and/or analyzed during the study available from the corresponding author on reasonable request.

## Abbreviations

COREQ: Consolidated criteria for Reporting Qualitative Research
KSPH: Kunshan People’s Hospital
WXSPH: Wuxi Second People’s Hospital
SHCH: Shanghai Children’s Hospital
NJPH: Northern Jiangsu People’s Hospital
AHYZU: Affiliated Hospital of Yangzhou University
